# A Phase I Study of the Oral Dual-Acting Pan-PI3K/mTOR Inhibitor Bimiralisib in Patients with Advanced Solid Tumors

**DOI:** 10.3390/cancers16061137

**Published:** 2024-03-13

**Authors:** Filip Janku, Grace M. Choong, Mateusz Opyrchal, Afshin Dowlati, Cinta Hierro, Jordi Rodon, Andreas Wicki, Martin D. Forster, Sarah P. Blagden, Jun Yin, Joel M. Reid, Helene Muller, Natasa Cmiljanovic, Vladimir Cmiljanovic, Alex A. Adjei

**Affiliations:** 1 The University of Texas MD Anderson Cancer Center, Houston, TX 77030, USA; fjanku@monterosatx.com (F.J.); jrodon@mdanderson.org (J.R.); 2 Mayo Clinic Rochester, Department of Oncology, Rochester, MN 55905, USA; choong.grace@mayo.edu (G.M.C.); reid@mayo.edu (J.M.R.); 3 Roswell Park Comprehensive Cancer Center, Buffalo, NY 14263, USA; mopyrch@iu.edu; 4 University Hospitals of Cleveland, Cleveland, OH 44106, USA; afshin.dowlati@uhhospitals.org; 5 Vall d’Hebron Institute of Oncology (VHIO), Vall d’Hebron University Hospital, 08035 Barcelona, Spain; chierro@iconcologia.net; 6 University Hospital Basel, 4031 Basel, Switzerland; andreas.wicki@usz.ch; 7 UCL Cancer Institute, University College London Hospitals NHS Trust, London NW1 2PG, UK; martin.forster@uclh.nhs.uk; 8 Early Phase Clinical Trials Unit, Department of Oncology, Oxford University Hospitals NHS Foundation Trust, Oxford OX3 7LJ, UK; sarah.blagden@oncology.ox.ac.uk; 9 Division of Clinical Trials and Biostatistics, Mayo Clinic, Rochester, MN 55905, USA; vivien.yin@moffitt.org; 10 PIQUR Therapeutics AG, 4051 Basel, Switzerland; helene.muller@swissrockets.com (H.M.); natasa.cmiljanovic@swissrockets.com (N.C.); vladimir.cmiljanovic@swissrockets.com (V.C.)

**Keywords:** PI3K/mTOR inhibitor, clinical trial, solid tumor, safety, intermittent dosing schedule

## Abstract

**Simple Summary:**

The phosphatidylinositol 3-kinase (PI3K)/Akt (protein kinase B)/mammalian target of the rapamycin (mTOR) signaling pathway is important in regulating cell proliferation, growth, metabolism, and motility in response to environmental and growth signals. Inhibition of the PI3K/Akt/mTOR pathway by specific targeted inhibitors has been shown to lead to regression in human tumors in the preclinical setting. Clinically, several drugs targeting this pathway are in development. However, some of them have been subsequently withdrawn due to dose-related toxicity issues. Bimiralisib is an oral-balanced dual-acting PI3K/mTOR inhibitor. The aim of our study was to assess safety with different schedules of administration of bimiralisib. Results showed a good safety profile for the drug when administered using intermittent schedules, which may extend the drug exposure of patients and potentially increase the chance to see antitumor efficacy. The improved safety profile of the drug when given on an intermittent schedule also supports future potential combination with other targeted therapies.

**Abstract:**

Background: Bimiralisib is a pan-PI3K/mTOR inhibitor demonstrating antitumor efficacy in preclinical models. The objectives of this study were to identify a maximum tolerated dose (MTD), pharmacokinetics (PK), a dosing schedule, and adverse events (AEs) in patients with advanced solid tumors. Patients and Methods: Patients received oral bimiralisib to determine the MTD of one continuous (once daily) and two intermittent schedules (A: Days 1, 2 weekly; B: Days 1, 4 weekly) until progression or unacceptable AEs occurred. Results: The MTD for the continuous schedule was 80 mg, with grade three fatigue as the dose-limiting toxicity (DLT). No MTD was reached with intermittent schedules, with only one DLT in schedule B. PK analysis suggested that 140 mg (schedule A) was within the biologically active dose range and was selected for further exploration. The most frequent treatment-emergent AEs were hyperglycemia (76.2%) in the continuous schedule, and nausea (56–62.5%) in schedules A and B. The most frequent treatment-emergent > grade three AE for all schedules combined was hyperglycemia (28.6%, continuous schedule; 12.0%, schedule A; 12.5%, schedule B). There was one partial response in a head and neck squamous cancer patient with a NOTCH1^T1997M^ mutation. Conclusions: Bimiralisib demonstrated a manageable AE profile consistent with this compound class. Intermittent schedules had fewer > grade three AEs, while also maintaining favorable PK profiles. Intermittent schedule A is proposed for further development in biomarker-selected patient populations.

## 1. Introduction

The phosphatidylinositol 3-kinase (PI3K)/Akt (protein kinase B)/mammalian target of the rapamycin (mTOR) signaling pathway is important in regulating cell proliferation, growth, metabolism, and motility in response to environmental and growth signals [1,2,3]. Deregulated PI3K/Akt/mTOR signaling is a key driver of proliferation in many human cancers and has been shown to contribute to drug resistance in many types of neoplasia [1,2,3,4,5,6]. The various members of the PI3K/mTOR/AKT signaling pathway display a variety of gain of function and loss of function mutations that, alone or in combination, are key drivers for cancer growth [1,2,3,4,5,6]. A prominent example is the PIK3CA gene that encodes p110α, which is commonly mutated or amplified in a variety of cancers. Frequent loss of function in the tumor suppressor gene phosphatase and tensin homolog (PTEN) is also observed [1,2,3,5,6]. As the PI3K/Akt/mTOR pathway plays a crucial role in cancer growth, its inhibition by specific targeted inhibitors has been shown to lead to regression in human tumors in the preclinical setting [2,4,7,8,9].

Clinically, many drugs targeting the PI3K/and/or the mTOR pathways as well as AKT are currently in development for both adult and pediatric populations [1,2,3,5,10,11]. The marketed rapalogs, everolimus and temsirolimus (mTOR inhibitors) [12,13], have been joined by PI3K delta inhibitors for hematological malignancies, including idelalisib (Zydelig^®^, GS1101, CAL101) [14,15] and duvelisib (Copiktra^®^, INK-1197; IPI-145; VS-0145) [16]. Moreover, Leniolisib (CDZ-173, Joenja^®^) was approved for APDS (activated PI3K delta syndrome) [17]. The pan-PI3K inhibitor copanlisib (Aliqopa^®^, BAY 80-6946) has also been approved for hematological malignancies [18], while in solid tumors the PI3K alpha inhibitor alpelisib (Piqray^®^, BYL719) has been approved for advanced or metastatic breast cancer [19,20] and as Vijoice^®^ (BYL719) for PROS (PI3K alpha-related overgrowth syndrome) [21]. Of the several AKT inhibitors in development, only capivasertib (Truqap^®^, AZD5363) combined with fulvestrant has been recently approved for adults with hormone receptor (HR)-positive, HER2-negative, locally advanced or metastatic breast cancer and one or more biomarker alterations in PIK3CA, AKT1, or PTEN [22]. Despite these successes, PI3K and mTOR inhibitors have shown some adverse effects in the clinic [23]. In particular, some of the PI3K delta inhibitors for the treatment of hematological malignancies have been subsequently withdrawn due to dose-related toxicity issues [24].

Bimiralisib is an oral-balanced dual-acting PI3K/mTOR inhibitor that selectively targets all four isoforms of class I PI3K (α, β, γ, δ) and mTOR. Bimiralisib strongly reduces phosphorylation of pAKTSer473 (IC50 between 0.05 μM to 0.20 μM), which correlates with strong inhibition of other PI3K signaling pathway components [7,8,25].

With respect to efficacy in preclinical models and in cancer patients, dual inhibition of PI3K and mTOR appears to be superior to the inhibition of either of the targets alone. However, this improved activity has been shown to be less tolerated in the clinic, preventing approval of a dual PI3K/mTOR inhibitor thus far. Despite these issues, several clinical programs are still ongoing, including the evaluation of the inhibitors bimiralisib, apitolisib, dactolisib, paxalisib, gedatolisib, samotolisib, and voxtalisib. Indeed, gedatolisib has recently been granted breakthrough therapy designation for the treatment of hormone receptor-positive/HER2-negative metastatic breast cancer [20].

Optimization of dosing schedules is essential in order to fully exploit the therapeutic potential of dual PI3K/mTOR inhibitors like bimiralisib. The study PQR309-003 was an open-label, multi-center, non-randomized, dose-escalation, Phase 1 study with the primary objective of determining the MTD for continuous and intermittent dosing schedules with a recommendation for use in further clinical trials. Safety, tolerability, pharmacokinetic (PK), and preliminary efficacy of bimiralisib was also evaluated in the treatment of patients with advanced solid tumors as per Phase 1 standards.

## 2. Materials and Methods

### 2.1. Patients

This Institutional Review Board/Ethics Committee-approved study was conducted between January 2015 and March 2019. All patients provided written informed consent to participate in the study prior to being screened. Patients aged ≥18 years, with an Eastern Cooperative Oncology Group (ECOG) performance status of 0–1, who were able to swallow an oral medication, with a diagnosis of a solid malignancy for which there was no standard, curative, or life-prolonging treatment available, and with a radiographically or clinically evaluable tumor, were eligible. Patients required adequate hematologic, renal, and hepatic function, which were defined as hemoglobin ≥ 9 g/dL; an absolute neutrophil count (ANC) ≥ 1500/mm^3^; a platelet count ≥ 100,000/mm^3^; calculated creatinine clearance ≥ 60 mL/min or serum creatinine < 1.5 ULN; bilirubin ≤ ULN or <1.5 × ULN if liver metastases or total 3 × ULN with direct bilirubin ≤ ULN in patients with well documented Gilbert Syndrome; and aspartate aminotransferase (AST), alanine aminotransferase (ALT), ≤2.5 × ULN (if elevation could be reasonably ascribed to the presence of metastatic disease to liver and/or bone) and hemoglobin A1c (HbA1c) ≤ 7%, and fasting plasma glucose (FPG) ≤ 7.0 mmol/L (125 mg/dL). All women of childbearing age had to have a negative pregnancy test prior to enrollment.

Patients were excluded if they had concurrent or previous anti-cancer chemotherapy, hormone therapy (except in prostate cancer), immunotherapy or investigational agents < 3 weeks, or palliative radiation < 2 weeks prior to the first day of study treatment. Patients with brain metastases were eligible if they received gamma knife irradiation > 2 weeks or whole brain radiation > 4 weeks before start of treatment and if intracranial disease was clinically stable. Patients who were on a short steroid regimen (≤1 week) or with a dose up to 1 mg dexamethasone daily equivalent were allowed in the study. Patients who were unable to take oral medications or adequately absorb the study drug (due to altered anatomy or concurrent medications) were not eligible. Patients with a history of cardiac disease with heart failure NYHA class III–IV, LVEF < 40%, myocardial infarction ≤ 6 months prior to enrolment, interstitial pneumonitis, chronic oxygen supplementation, or significant mood disorder confirmed with PHQ-9 score ≥ 12 or GAD-7 score ≥ 15 were also excluded.

### 2.2. Study Design

This was a Phase 1, open-label, multi-center, non-randomized, dose escalation study evaluating DLTs, tolerability, PK, and preliminary efficacy of bimiralisib in the treatment of patients with advanced solid tumors for which no standard curative or life-prolonging therapy is available. This study was conducted in the US (MD Anderson Cancer Center, Houston; Mayo Clinic Rochester; Roswell Park Comprehensive Cancer Center, Buffalo; University Hospitals of Cleveland), Spain (Vall Hebron University Hospital), Switzerland (Basel University Hospital), and UK (University College London Hospitals NHS Trust and Oxford Universities NHS Trust).

The primary objective of the study was to determine the MTD of bimiralisib administered according to three different dosing schedules. Bimiralisib was formulated as 20 mg and 80 mg capsules for oral administration. The first dosing schedule to be evaluated was continuous (once daily) dosing over a 21-day cycle. Following the results of pre-clinical data suggesting that continuous exposure to bimiralisib may not be necessary to achieve full efficacy [7], two intermittent dosing schedules were evaluated in parallel: intermittent schedule A, with bimiralisib administered for two consecutive days each week followed by five days without treatment (“2 days on/5 days off”); and intermittent schedule B, with bimiralisib administered on Day 1 and Day 4 (“Monday/Thursday”) of each week. Patients enrolled in the continuous schedule and intermittent schedule A received a single lead-in dose, administered on Day 3 prior to the start of the first 21-day treatment cycle, to characterize single-dose pharmacokinetics. Based on the previous first-in-human trial with bimiralisib [26], together with the expected toxicity profile of PI3K/mTOR inhibitors, the starting dose (dose-level 1) of bimiralisib in Study PQR309-003 was set at 80 mg daily.

Enrollment of cohorts into the dose escalation followed a typical 3 + 3 design, with escalation/de-escalation based on the dose-limiting toxicities observed as described in Table 1 and Table 2. Up to 5 dose levels were planned in the continuous schedule to a maximum of 180 mg daily. For the intermittent schedules, up to 7 dose levels were planned, to a maximum of 240 mg daily. One dose level for de-escalation was planned for all schedules. Patient cohorts, consisting initially of three patients, were enrolled depending on the safety and tolerability of the initial cohort. Patients were treated until disease progression, unacceptable toxicity, patient’s request for withdrawal, investigator judgment, or death, whichever came first.

### 2.3. Assessments

Safety was monitored by physical examination, vital signs, weight, electrocardiogram, ECOG performance status, laboratory evaluations (including glucose monitoring), and patient self-reported mood questionnaires, as well as AEs. All AEs were coded with NCI Common Terminology Criteria for Adverse Events (CTCAE) v4.03 [27]. The MTD was defined as the maximum dose level at which ≤1/6 patients have DLTs. A DLT was defined by the occurrence of grade 3 or 4 AE that is possibly, probably, or definitely related to bimiralisib within the first 21 days of treatment. Bimiralisib treatment was interrupted if patients experienced any DLTs. If the patient derived benefit from the treatment as determined by the investigator, treatment could be resumed at a lower dose in agreement with the study sponsor.

Patients were discontinued from trial if they had evidence of clinical, symptomatic, or radiographic disease progression or relapse, unacceptable drug related AEs, non-compliance to the protocol, voluntary withdrawal, or pregnancy. Treatment compliance was established with patient diaries and records of medication used and dosages administered.

Efficacy of treatment was determined by measuring reported lesions at baseline and after every 2 cycles. RECIST 1.1 criteria [28] were used to assess objective tumor response (complete response [CR], partial response [PR], stable disease [SD], progressive disease [PD]) [28].

Blood samples were collected to enable full PK profiling of bimiralisib. The PK samples were taken pre-dose and at 0.5, 1, 2, 4, 6, 8, and 24 h post-dosing in the three schedules, with additional timepoints depending on the schedule (48 h in the continuous schedule, 12, 16, and 48 h in intermittent schedule A and 12, 16, and 72 h in intermittent schedule B). PK sampling was repeated on cycle 1 Day 8 (continuous schedule: pre-dose and 1 h post-dose; intermittent schedules A and B: pre-dose and 0.5, 1, 2, 4, 6, 8, and 24 h post-dose), on cycle 1 Day 15 (continuous schedule: pre-dose and 0.5, 1, 2, 4, 6 and 8 h post-dose; intermittent schedules A and B: pre-dose and 4 h post-dose), and on cycle 2 Day 1 (continuous dosing: pre-dose and 1 h post-dose). Samples were sent to a central laboratory for analysis using liquid-chromatography mass spectrometry [26]. The pharmacokinetics of bimiralisib were determined by non-compartmental analysis. Area under the concentration vs. time curve (AUC) was calculated using the linear up log down trapezoidal method [29]. Accumulation was calculated as the ratio of cycle 1 on Day 15 AUC_0–8h_ versus cycle 1 on Day -3 AUC_0–8h_. The apparent half-life (t1/2) was calculated from the following equation: t1/2 = −(0.693 × τ)/ln((R − 1)/R), where R is accumulation ratio and τ is the dosing interval (24 h).

### 2.4. Statistics

Descriptive statistics were used to present the data. Categorical data were summarized in contingency tables presenting frequencies and percentages. Continuous data were summarized using the number of non-missing values (n), mean, standard deviation (SD), median, Q1, Q3, minimum and maximum values, as appropriate. Data were analyzed using SAS. No tests of significance were planned to compare study treatment groups on baseline data. The efficacy and safety analysis set comprised all patients who receive ≥1 dose of bimiralisib.

## 3. Results

### 3.1. Patient Demographics and Characteristics

A total of 70 patients were enrolled in this study with 21 patients (30.0%) enrolled to continuous dosing, 25 patients (35.7%) to intermittent schedule A and 24 patients (34.3%) to intermittent schedule B. The majority of patients were female (*n* = 49, 70%) and white (*n* = 65, 92.9%) with a median age of 57 years (range: 19–83 years). The most common tumor types were ovarian/gynecologic cancer (*n* = 14), breast cancer (*n* = 11), sarcoma/other (*n* = 8), and colorectal cancer (*n* = 7). The best response to their last prior cancer therapies was progressive disease (50%) and stable disease (25.7%). The mean time since initial diagnosis was 67.8 months (SD 63.8). Most patients (*n* = 54, 77.2%) had received three or more prior lines of cancer therapies. Additional demographics and baseline patient characteristics are found in Table 3.

### 3.2. Dose Escalation, DLTs, and MTD Determination

Dose-escalation in the continuous dosing schedule started with 6 patients treated at 80 mg daily with no DLT observed. At the next dose level of 120 mg daily, DLTs (grade 3 fatigue) were seen in three of five patients. Five patients were, therefore, recruited to the 100 mg daily cohort. Four of these five patients had dose interruptions or discontinued program due to treatment-related AEs during their first 21-day dosing cycle, with one of them experiencing a DLT (grade 3 fatigue), indicating that this dose was also not tolerable. Therefore, five additional patients were enrolled in the continuous dosing schedule at 80 mg daily (leading to 11 patients treated at 80 mg daily in total) and this dose was declared as MTD. Intermittent schedules A and B were initiated thereafter in parallel. There was no DLT observed in the intermittent schedule A up to 200 mg and one DLT was seen in the intermittent schedule B at the 100 mg dose level (clinically asymptomatic grade 4 lipase elevation) (Table 4 and Table 5). Since neither intermittent schedule reached MTD, 140 mg on schedule A was selected for further exploration as it demonstrated the most favorable safety and PK profile.

Dose interruptions and dose reductions due to treatment-emergent AEs (TEAEs) were most common in the continuous schedule (dose interruption: *n* = 11, 52%; dose reduction: *n* = 2, 10%) compared to the intermittent schedule A (dose interruption: *n* = 9, 36%; dose reduction: *n* = 0, 0%) and intermittent schedule B (dose interruption: *n* = 9, 38%; dose reduction: *n* = 1, 4%).

The mean duration of treatment (and standard deviations) at the recommended doses of each schedule was 57.2 ± 74.1 days at 80 mg continuous dosing, 72.7 ± 39.0 days at 140 mg intermittent schedule A and 82.7 ± 81.5 days at 140 mg intermittent schedule B. Patients discontinued study treatment due to the following reasons: death unrelated to study drug (vascular rupture) (*n* = 1, 1.4%), radiological progressive disease (*n* = 40, 57.1%), AEs (*n* = 9, 12.9%), investigator decision (*n* = 6, 8.6%), sponsor decision (*n* = 1, 1.4%), withdrawal of consent (*n* = 12, 17.1%), and clinical disease progression (*n* = 1, 1.4%). Of nine patients who discontinued the study due to AEs (5 in continuous dosing, 1 in intermittent schedule A, and 3 in intermittent schedule B), five had AEs considered to be treatment-related (fatigue (2 in continuous dosing and 1 in schedule B) and ALT/AST increased, myalgia, polyuria, pollakiuria (all in schedule B)) (Table 6).

### 3.3. Pharmacokinetics of Bimiralisib

Bimiralisib plasma concentration-time data were available for all 70 patients in all three dosing schedules. PK data are summarized in Table 7. After the first dose of bimiralisib, the median time to reach the peak plasma concentration (Tmax) was 2 h (range, 0.5–48 h) and was comparable across all three dosing schedules. The increase in concentration-time data was not proportional to doses and a high variability between subjects was observed in all dosing schedules. The estimate of the half-life was calculated based on bimiralisib accumulation on the continuous schedule. The median accumulation ratio and half-life values for patients who had AUC_0–8h_ data for cycle 1 on Day -3 and cycle 1 on Day 15 were 2.9 (range, <1.0–9.3) and 39.5 h (range < 6–146 h). A less than linear dose-exposure relationship was observed at intermittent doses higher than 140 mg. Hence, 140 mg daily on an intermittent schedule was chosen for subsequent studies.

### 3.4. Safety of Bimiralisib

Nearly all patients (*n* = 69; 99%) experienced one or more TEAEs, most of which were related to the study drug (*n* = 63; 91%) (Table 4).

Most frequently reported TEAEs, regardless of causality, included nausea, fatigue, hyperglycemia, decreased appetite, diarrhea, vomiting, weight decrease, dry mouth, anxiety, constipation, rash, back pain, disturbance in attention, pruritus, AST increased, asthenia, disease progression, and dry skin (Table 4).

Grade > 3 TEAEs were experienced by 67.1% of patients (81% in continuous dosing, 56% in intermittent schedule A, and 66.7% in intermittent schedule B). The most frequent grade > 3 TEAEs included hyperglycemia (28.6% in continuous dosing, 12% in intermittent schedule A, and 12.5% in intermittent schedule B) and fatigue (33.3% in continuous dosing, 0% in intermittent schedule A, and 8.3% in intermittent schedule B).

Twenty-eight patients (40%) reported one or more serious adverse events (SAEs). The most frequently reported SAEs were disease progression and fatigue. Four (6%) of these SAEs were considered related to study drug; two (fatigue) in continuous dosing, zero in intermittent schedule A, and two (diarrhea and hyperglycemia) in intermittent schedule B. These events are consistent with the known safety profile of bimiralisib [26] and other drugs in its class.

### 3.5. Efficacy

**Figure 1:**
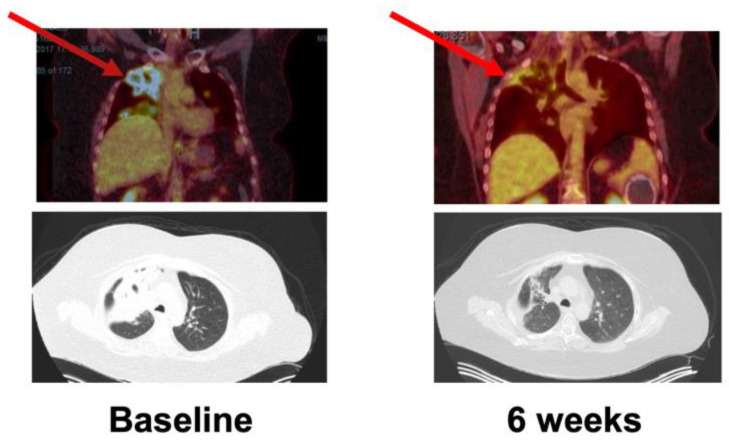
Partial response in patient with a head and neck squamous cancer and loss of function NOTCH1T1997M mutation. A partial response in a patient with metastatic HNSCC and NOTCH1T1997M loss of function mutation demonstrated on PET/CT and obtained after 6 weeks of therapy compared to baseline. The patient received the intermittent dosing schedule A (200 mg). 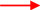: Target lesion.

**Figure 2:**
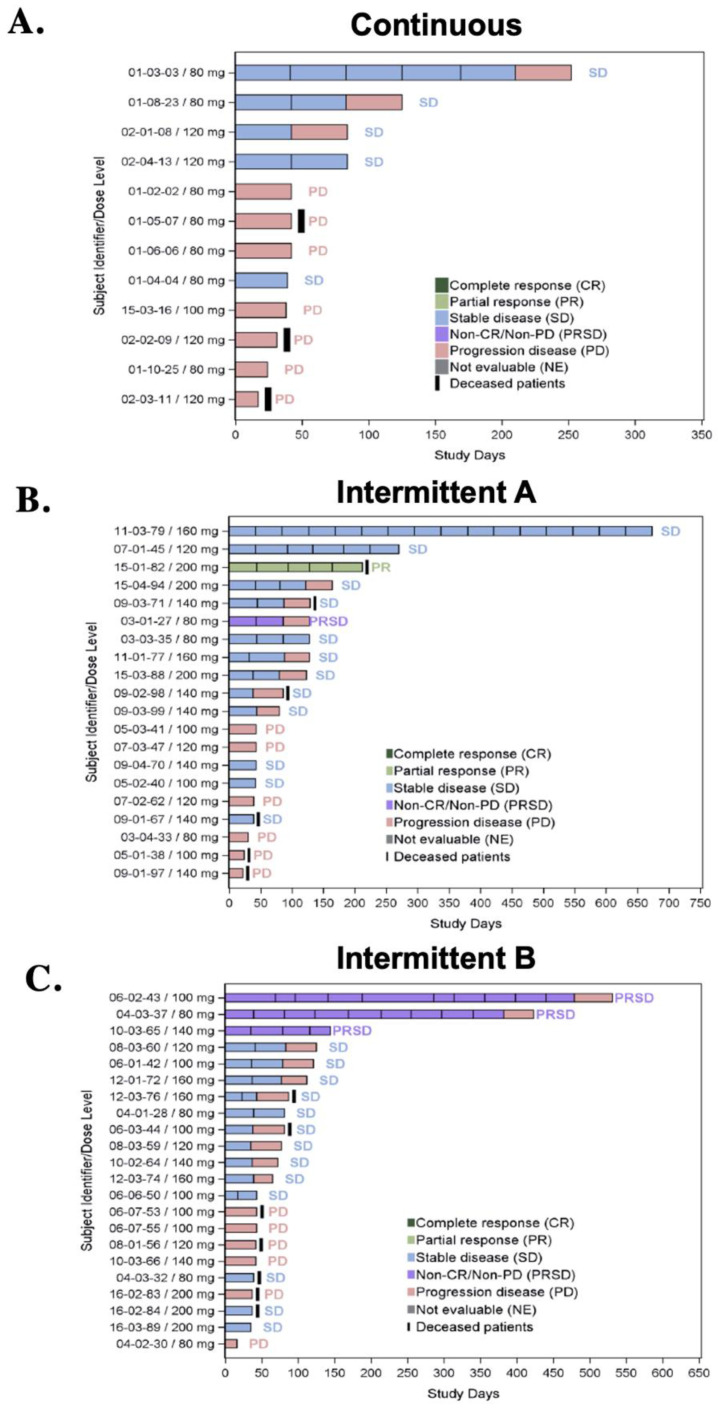
Best overall response per patient per dosing schedule. Efficacy and time on therapy for (**A**) continuous schedule; (**B**) intermittent schedule A; and (**C**) intermittent schedule B. Patients with non-measurable disease per RECIST 1.1, who had non-CR/non-PD are indicated as PRSD.

The best overall responses for continuous and intermittent dosing schedules A and B are depicted in Table 8. No patient achieved CR. A PR (85% reduction in sum of target lesions per RECIST 1.1) was observed as the best overall response in a patient (1.4%) with a head and neck squamous cancer and loss of function NOTCH1T1997M mutation, receiving the intermittent schedule A at a dose of 200 mg. The time to response was 45 days and the duration of response was 8.4 months (Figure 1). This patient passed away from events unrelated to the study drug or disease progression. SD was observed in 30 (43.0%) patients (23.8% in continuous dosing, 48.0% in intermittent schedule A, and 54.2% in intermittent schedule B, Figure 2).

A total of 19 (27.1%) patients died during the study, 16 due to disease progression and 3 due to other reasons unrelated to bimiralisib, according to the study investigators (vessel rupture, sepsis, new small cell carcinoma).

## 4. Discussion

In this study, 70 patients with advanced solid tumors were enrolled and received a continuous or one of two intermittent bimiralisib dosing schedules. Overall, the safety profiles across the three treatment schedules were comparable to previous data with daily administration of bimiralisib [30] and other compounds in the class and were manageable. However, the intermittent schedules, in particular schedule A, were better tolerated while maintaining favorable PK profiles. Albeit with limited numbers of patients, the intermittent regimen A had lower numbers in important safety categories, such as any drug-related grade > 3 AEs and SAEs, especially hyperglycemia (12%) and fatigue (0%), as well as TEAE-related dose interruptions, reductions, and discontinuations. The intermittent regimen A at the dose of 140 mg bimiralisib on days one and two weekly was therefore selected as the preferred regimen for further clinical testing. This is in accordance with previously published data suggesting a potential for intermittent PI3K inhibitor dosing schedules (reviewed in [5]). For instance, an early-phase study of the PI3K alpha-selective inhibitor TAK-117 demonstrated that intermittent dosing has an acceptable safety profile and enables higher doses and total weekly exposures as compared to daily dosing [31].

The limited clinical efficacy observed in this study is most likely the result of a patient population without molecular selection, as has been recommended. At the time of trial initiation in 2015, limited clinical data were available regarding the potential to select patients for treatment with PI3K/mTOR/AKT inhibitors based on pathway alterations/mutations in tumor tissue at baseline. Hence, trial design followed the “classical” Phase 1 concept where safety and definition of MTD were the primary goals tested in a broader advanced solid-tumor patient population. Definition of an appropriate dose and administration schedule for further evaluation in indication-specific trials was the principal aim. Although clinical exploitation of PI3K pathway aberrations, most notably PIK3CA-mutation status, has since proven to be successful in selecting advanced breast cancer patients for treatment with the PI3K alpha-selective inhibitor alpelisib [19] and the AKT inhibitor capivasertib [22] in combination with fulvestrant, elucidation of novel patient-selection approaches in solid-tumor patients remains challenging. In this context, a durable PR in a patient with head and neck squamous cancer characterized by a loss of function NOTCH1T1997M mutation treated with the intermittent regimen A was of particular interest [32]. Indeed, preclinically, NOTCH1 loss of function mutations were found to predict response of head and neck squamous cancer models to PI3K/mTOR inhibition, prompting the suggestion that this could constitute a rationale for a biomarker-driven targeted therapeutic approach [33]. Consequently, this was the basis for a small exploratory trial in eight patients with head and neck squamous cancer and loss of function NOTCH1T1997M mutations treated with the intermittent bimiralisib regimen, which demonstrated one confirmed PR and three SDs [32]. Based on these initial signals, further exploration of bimiralisib in this patient population is planned. An expanded biomarker analysis for additional markers of pathway dependency may also contribute to optimizing patient selection, as well as selection of additional tumor types for clinical evaluation.

Taken together, these observations support further biomarker-guided development with the intermittent regimen, including appropriate combinations to improve efficacy and address resistance mechanisms.

## 5. Conclusions

The oral PI3K/mTOR inhibitor bimiralisib demonstrated a manageable and potentially improved safety profile when administered using an intermittent schedule. The dose of 140 mg bimiralisib on days one and two weekly was selected as the regimen for further clinical testing. Based on the efficacy signal seen in a patient with head and neck squamous cell carcinoma with a loss of function NOTCH1 mutation, a biomarker-driven strategy is now being pursued.

## Figures and Tables

**Table 1:** Continuous Dosing Schedule.

Dose Level	Bimiralisib *
−1 **	60 mg daily
1 (starting)	80 mg daily
2	120 mg daily
3	140 mg daily
4	160 mg daily
5	180 mg daily

* Patients receive a single dose on Day -3 (lead-in dose) followed by daily dosing. ** Dose-level −1 tested only if DLTs occur in ≥2 patients at dose-level 1.

**Table 2:** Intermittent Dosing Schedule.

Intermittent Schedules A and B Dose Level	Bimiralisib *
1 (starting)	MTD qd of continuous schedule
2	MTD qd + 20 mg
3	MTD qd + 40 mg
4	MTD qd + 60 mg
5	MTD qd + 80 mg

* Dose escalation allowed as high as possible in 20 mg increments. After having established dose-level 5 (MTD qd + 80 mg) as safe, further dose escalations can be performed with up to 40 mg increments. This would result in a dose of up to 200 mg for dose-level 6, up to 240 mg for dose-level 7 and so on for both intermittent schedules A and B until the MTD for the respective intermittent schedules are reached. Where the dose escalation is performed in increments of 40 mg and 2 or more DLTs are observed, the next dose level evaluated may be 20 mg lower.

**Table 3:** Baseline patient demographics and disease characteristics.

Characteristic	Continuous (*n* = 21)	Intermittent A (*n* = 25)	Intermittent B (*n* = 24)	Total (*n* = 70)
Median age, years (range)	58 (41–78)	56 (22–81)	56 (19–83)	57 (19–83)
Female, *n* (%)	14 (66.7)	17 (68)	18 (75)	49 (70)
Male, *n* (%)	7 (33.3)	8 (32)	6 (25)	21 (30)
Race, *n* (%)				
White	20 (95.2)	22 (88)	23 (95.8)	65 (92.9)
Black or African American	1 (4.8)	2 (8)	0 (0)	3 (4.3)
Asian	0 (0)	1 (4)	1 (4.2)	2 (2.9)
ECOG performance status, *n* (%)				
0	9 (42.9)	9 (36)	9 (37.5)	27 (38.6)
1	12 (57.1)	16 (64)	15 (62.5)	43 (61.4)
Smoking history, *n* (%)				
Yes	11 (52.4)	11 (44)	12 (50)	34 (48.6)
No	10 (47.6)	15 (56)	12 (50)	36 (51.4)
Disease primary diagnosis, *n* (%)				
Lung	3	4	0	7
Breast	1	6	4	11
Prostate/GU	2	0	1	3
Ovarian/Gynecologic	5	5	4	14
Gastroesophageal	2	0	0	2
Colorectal	2	3	2	7
Pancreatic	1	0	0	1
Anal	1	1	1	3
Sarcoma/other	4	2	2	8
Melanoma	0	1	0	1
Lymphoma	0	2	0	2
GBM	0	0	1	1
Disease stage at diagnosis, *n* (%)				
Stage III	4 (19)	4 (16)	2 (8.4)	10 (14.3)
Stage IV	4 (19)	7 (28)	6 (25)	17 (24.3)
Disease stage at study entry, *n* (%)				
Stage III/IV	3 (14.3)	7 (28)	1 (4.2)	11 (15.7)
Recurrent	0 (0)	4 (16)	5 (20.8)	9 (12.9)
Metastatic	18 (85.7)	14 (56)	18 (75)	50 (71.4)
Prior therapy, *n* (%)				
Prior surgery or non-radiation procedure	19 (90.5)	18 (72)	15 (62.5)	42 (60)
Prior radiation	10 (47.6)	16 (64)	17 (70.8)	43 (61.4)
Prior antineoplastic therapy				
1	1 (4.8)	4 (16)	3 (12.5)	8 (11.4)
2	2 (8.5)	2 (8)	4 (16.7)	8 (11.4)
≥3	18 (85.7)	19 (76)	17 (70.8)	54 (77.2)
Best response to last prior antineoplastic therapy, *n* (%)				
Partial response	0 (0)	6 (24)	3 (12.5)	9 (12.9)
Stable disease	9 (42.9)	6 (24)	3 (12.5)	18 (25.7)
Progressive disease	12 (57.1)	11 (44)	12 (50)	35 (50.0)
Unknown	0	2 (8)	6 (25)	8 (11.4)

**Table 4:** Adverse Events.

AE, *n* (%)	Continuous (*n* = 21)	Intermittent A (*n* = 25)	Intermittent B (*n* = 24)	Total *n* = 70
Any TEAE	21 (100)	24 (96)	24 (100)	69 (98.9)
Any drug-related AE	21 (100)	22 (88)	21 (88)	63 (91)
Common TEAE (≥10% of patients)				
Nausea	12 (57.1)	14 (56)	15 (62.5)	41 (58.6)
Fatigue	13 (61.9)	12 (48)	10 (41.7)	35 (50.0)
Hyperglycemia	16 (76.2)	6 (24)	9 (37.5)	31 (44.3)
Decreased appetite	11 (52.4)	9 (36)	8 (33.3)	28 (40.0)
Diarrhea	10 (47.6)	11 (44)	7 (29.2)	28 (40.0)
Vomiting	10 (47.6)	5 (20)	7 (29.2)	22 (31.4)
Weight decrease	11 (52.4)	4 (16)	3 (12.5)	18 (25.7)
Dry Mouth	10 (47.6)	3 (12)	4 (16.7)	17 (24.3)
Anxiety	5 (23.8)	5 (20)	6 (25)	16 (22.9)
Constipation	3 (14.3)	3 (12)	8 (33.3)	14 (20.0)
Rash	4 (19.1)	5 (20)	5 (20.8)	14 (20.0)
Back pain	4 (19.1)	4 (16)	5 (20.8)	13 (18.6)
Disturbance attention	11 (52.4)	1 (4)	1 (4.2)	13 (18.6)
Pruritus	6 (28.6)	2 (8)	5 (20.8)	13 (18.6)
AST elevation	3 (14.3)	4 (16)	5 (20.8)	12 (17.1)
Asthenia	6 (28.6)	2 (8)	4 (16.7)	12 (17.1)
Disease progression	3 (14.3)	5 (20)	3 (12.5)	11 (15.7)
Dry skin	7 (33.3)	3 (12)	1 (4.2)	11 (15.7)
Headache	0 (0)	3 (12)	7 (29.2)	10 (14.3)
Hyperinsulinemia	7 (33.3)	1 (4)	1 (4.2)	9 (12.9)
Hypokalemia	6 (28.6)	1 (4)	2 (8.3)	9 (12.9)
Abdominal pain	1 (4.76)	4 (16)	3 (12.5)	8 (11.4)
Anemia	0 (0)	3 (12)	5 (20)	8 (11.4)
Dyspepsia	1 (4.76)	3 (12)	4 (16.7)	8 (11.4)
ALT elevation	1 (4.76)	3 (12)	3 (12.5)	7 (10)
Cough	2 (9.5)	3 (12)	2 (8.3)	7 (10)
Dehydration	6 (28.6)	1 (4)	0 (0)	7 (10)
Depression	5 (23.8)	0 (0)	2 (8.3)	7 (10)
Dizziness	3 (14.3)	1 (4)	3 (12.5)	7 (10)
Dyspnea	4 (19.1)	2 (8)	1 (4.2)	7 (10)
Muscular weakness	3 (14.3)	0 (0)	4 (16.7)	7 (10)
Peripheral edema	3 (14.3)	2 (8)	2 (8.3)	7 (10)
Tachycardia	4 (19.1)	2 (8)	1 (4.2)	7 (10)
Dysgeusia	4 (19.1)	0 (0)	2 (8.3)	6 (8.6)
Irritability	0 (0)	3 (12)	2 (8.3)	5 (7.1)
Upper respiratory tract infection	1 (4.76)	3 (12)	1 (4.2)	5 (7.1)
Cachexia	4 (19.1)	0 (0)	0 (0)	4 (5.7)
Hyperkalemia	0 (0)	3 (12)	1 (4.2)	4 (5.7)
Hyperphosphatasemia	1 (4.76)	3 (12)	0 (0)	4 (5.7)
Malaise	4 (19.1)	0 (0)	0 (0)	4 (5.7)
Ascites	0 (0)	3 (12)	0 (0)	3 (4.3)
Confusional state	3 (14.3)	0 (0)	0 (0)	3 (4.3)
Early satiety	3 (14.3)	0 (0)	0 (0)	3 (4.3)
Hallucination	3 (14.3)	0 (0)	0 (0)	3 (4.3)
Mucosal Inflammation	0 (0)	3 (12)	0 (0)	3 (4.3)
Neck pain	0 (0)	0 (0)	3 (12.5)	3 (4.3)
Any grade ≥ 3 TEAE	17 (81)	14 (56)	16 (66.7)	47 (67.1)
Grade ≥ 3 TEAEs (≥5% of patients)				
Hyperglycemia	6 (28.6)	3 (12)	3 (12.5)	12 (17.1)
Fatigue	7 (33.3)	0 (0)	2 (8.3)	9 (12.9)
AST elevation	3 (14.3)	1 (4)	1 (4.2)	4 (5.7)
ALT elevation	1 (4.8)	1 (4)	2 (8.3)	4 (5.7)
Dehydration	4 (19)	0 (0)	0 (0)	4 (5.7)
Any serious AE	9 (43)	8 (32)	11 (46)	28 (40)
Any drug-related serious AE	2 (10)	0 (0)	2 (8)	4 (5.7)
Treatment-related death	0 (0)	0 (0)	0 (0)	0 (0)
Any drug-related grade ≥ 3 AE	11 (52.4)	5 (20)	10 (41.7)	26 (37.1)

**Table 5:** Dose-limiting toxicities and grade per dosing schedule and dose level.

Dosing Schedule	Dose Level	Patients Enrolled	No. of Patients with ≥1 DLT Events	Grade of DLT	TEAE Resulting in Dose Interruption	TEAE Resulting in Dose Reduction
Continuous Dosing	80 mg	11 *	0 (0%)	NA	4 (36%)	0 (0%)
	100 mg	5	1 (20%)	Grade 3 fatigue	3 (60%)	1 (20%)
	120 mg	5	3 (60%)	Grade 3 fatigue	4 (80%)	1 (20%)
	Total per schedule	21	4 (19%)	NA	11 (52%)	2 (10%)
Intermittent Schedule A	80 mg	4	0 (0%)	NA	1 (25%)	0 (0%)
	100 mg	3	0 (0%)	NA	1 (33%)	0 (0%)
	120 mg	4	0 (0%)	NA	1 (25%)	0 (0%)
	140 mg	6	0 (0%)	NA	2 (33%)	0 (0%)
	160 mg	3	0 (0%)	NA	1 (33%)	0 (0%)
	200 mg	5	0 (0%)	NA	3 (60%)	0 (0%)
	Total per schedule	21	0 (0%)	NA	9 (36%)	0 (0%)
Intermittent Schedule B	80 mg	4	0 (0%)	NA	2 (50%)	0 (0%)
	100 mg	7	1 (14.3%)	Grade 4 Lipase elevation	3 (43%)	0 (0%)
	120 mg	3	0 (0%)	NA	0 (0%)	0 (0%)
	140 mg	3	0 (0%)	NA	2 (67%)	1 (33%)
	160 mg	3	0 (0%)	NA	1 (33%)	0 (0%)
	200 mg	4	0 (0%)	NA	1 (25%)	0 (0%)
	Total per schedule:	24	1 (4.2%)	NA	9 (38%)	1 (4%)

NA: Not applicable. * Due to intolerable DLTs at dose ≥ level 100 mg, subsequent patients enrolled in the study were only given 80 mg daily dose.

**Table 6:** Study discontinuation due to adverse event.

AE, *n* (%)	Continuous (*n* = 21)	Intermittent A (*n* = 25)	Intermittent B (*n* = 24)	Total *n* = 70
Patients discontinuing study due to AE	5 (24.0%)	1 (4.0%)	3 (12.5%)	9 (12.9%)
Fatigue	3 (14.3%)		1 (4.2%)	4 (5.7%)
ALT Increased			2 (8.3%)	2 (2.8%)
Dehydration	2 (9.5%)			2 (2.8%)
AST Increased			1 (4.2%)	1 (1.4%)
Ejection Fraction decreased	1 (4.8%)			1 (1.4%)
Musculoskeletal chest pain			1 (4.2%)	1 (1.4%)
Myalgia			1 (4.2%)	1 (1.4%)
Polyuria			1 (4.2%)	1 (1.4%)
Pollakiuria			1 (4.2%)	1 (1.4%)
Renal Failure		1 (4.0%)		1 (1.4%)
Tumor pain	1 (4.8%)			1 (1.4%)
Urinary Tract Infection		1 (4.0%)		1 (1.4%)

**Table 7:** Pharmacokinetic data for bimiralisib at doses 80–200 mg over all three dosing schedules.

Dose Level (mg)	Period	Patients (*n*)	T_max_ (h) Median, (Range)	C_max_, (ng/mL) Mean (%CV)	AUC_0–8_, (h·ng/mL) Geometric Mean (%CV)	AUC_0–24_, (h·ng/mL) Geometric Mean (%CV)
Continuous Dosing						
80	Day -3	11	2 (0.5–24)	407 (47)	1530 (48)	4290 (50)
	Day 15	7	1 (0.5–8)	766 (60)	4090 (64)	NA
100	Day 1	5	1.7 (1–8)	441 (49)	1690 (29)	4660 (24)
	Day 15	2	2 (2–2)	1110 (ND)	5750 (ND)	NA
120	Day 1	5	2 (1–48)	876 (79)	2990 (65)	7600 (52)
	Day 15	1	8	982 (ND)	6430 (ND)	NA
Intermittent Dose Schedule A						
80	Day -3	4	1.5 (1–2)	410 (50)	1580 (53)	3540 (59)
	Day 8	3	1 (1–1)	614 (47)	2350 (10)	6740 (*n* = 1)
100	Day -3	4	1 (1–1)	543 (70)	1970 (59)	4170 (52)
	Day 8	3	1 (1–2)	966 (10)	3910 (22)	12,400 (*n* = 2)
120	Day -3	4	3 (1–4)	549 (44)	2590 (54)	5590 (62)
	Day 8	3	1 (1–24)	872 (79)	3710 (78)	8940 (69)
140	Day -3	6	1 (1–4)	709 (60)	2580 (48)	5810 (42)
	Day 8	6	1 (0.5–2)	923 (48)	3220 (28)	7590 (30; *n* = 3)
160	Day -3	3	1 (1–2)	539 (25)	2480 (16)	5830 (20)
	Day 8	3	2 (1–2)	857 (28)	4080 (41)	11,100 (64)
200	Day -3	4	2 (1–2)	1030 (34)	3480 (37)	7050 (37)
	Day 8	4	2 (1–4)	963 (61)	4460 (56)	15,900 (*n* = 2)
Intermittent Dose Schedule B						
80	Day 1	4	3 (1–6)	341 (17)	1680 (40)	4000 (55)
	Day 8	4	1.5 (1–8)	681 (30)	2600 (34)	6350 (44; *n* = 3)
100	Day 1	7	2 (0.5–8)	562 (98)	1920 (67)	4520 (54)
	Day 8	6	1.5 (0.5–6)	714 (69)	2880 (54)	6200 (67; *n* = 4)
120	Day 1	3	1 (1–2)	471 (35)	1530 (5)	3340 (4)
	Day 8	3	1 (1–8)	594 (34)	2210 (8)	5850 (24)
140	Day 1	3	1 (0.5–1)	1010 (37)	4150 (63)	9740 (68)
	Day 8	3	1 (1–2)	1560 (30)	5160 (44)	12,600 (*n* = 2)
160	Day 1	3	12 (1–12)	317 (63)	1050 (54)	3700 (29)
	Day 8	3	8 (2–22.3)	325 (83)	1770 (97)	ND
200	Day 1	4	1.5 (1–8)	734 (66)	3190 (72)	8010 (68)
	Day 8	4	1.5 (1–6)	1200 (46)	5800 (38)	14,700 (58; *n* = 3)

%CV: coefficient of variation; NA: not applicable; ND: not determined; T_max_: time point at which maximum concentration was reached; C_max_: maximum concentration based on plasma concentration; AUC_0–8_: AUC measured from 0–8 h; AUC_0–24_: AUC measured from 0–24 h.

**Table 8:** Best overall clinical response per dosing schedule.

Best Overall Response (BOR)	Treatment Group/Number of Patients			
	Continuous	Intermittent A	Intermittent B	All Schedules
	*n* = 21	*n* = 25	*n* = 24	*n* = 70
Complete response (CR)	0	0	0	0
Partial response	0	1 (4%)	0	1 (1.4%)
Stable disease	5 (23.8%)	12 (48%)	13 (54.2%)	30 (43%)
Non-CR/Non-PD	0	1 (4%)	3 (12.5%)	4 (5.7%)
Progressive disease (PD)	7 (33.3%)	6 (24%)	6 (25%)	19 (27.1%)
Missing	9 (42.9%)	5 (20%)	2 (8.3%)	16 (22.9%)

BOR: best overall response; CR: complete response; PD: progressive disease. Missing: imaging studies not performed at time of study discontinuation and response could not be assessed.

## Data Availability

The datasets used and/or analyzed during the current study are available from the corresponding author upon reasonable request.
